# Development and validation of soil test crop response model for beetroot (*Beta vulgaris*) grown in ultisols of India

**DOI:** 10.3389/fpls.2024.1481882

**Published:** 2025-01-28

**Authors:** Ramasamy Arulmani, Kuppayevalasu Malayappagounder Sellamuthu, Subramaniam Maragatham, Alargarsamy Senthil, Seenapuram Palaniswami Thamaraiselvi, Palaniappan Malathi, Govindaraja Sridevi

**Affiliations:** ^1^ Tamil Nadu Agricultural University, Coimbatore, India; ^2^ Department of Soil Science and Agricultural Chemistry, Coimbatore, India; ^3^ Department of Crop Physiology, Coimbatore, India; ^4^ Department of Floriculture and Landscape Architecture, Coimbatore, India

**Keywords:** beetroot, fertilizer prescription, STCR-IPNS, targeted yield model, NPK uptake, BCR

## Abstract

Soil Test Crop Response (STCR), a combined plant nutrient management system that enables to develop fertilizer prescription equations for balanced crop nutrition, higher yield, profitability, and better nutrient efficiency. Field trial was carried out on Typic Haplohmult soil of Nilgiris, Tamil Nadu during 2023-2024 by Implementing an Inductive combined with a targeted yield model. Field trial includes a gradient experiment with a green viz., *Chenopodium album*; a test crop experiment with beetroot (Hybrid Improved Crystal) and a validation experiment with beetroot. First, the fertility gradient was ensured by the biomass yield and soil fertility. Then, test crop experiment with beet root were conducted in the same field to derive the basic parameters viz., Nutrient Requirement (NR), contribution of nutrients from fertilizers (C_f_), contribution of nutrients from soil (C_s_) and contribution of nutrients from the Farm Yard manure (C_fym_). Using the basic parameters, fertilizer prescription equations were developed based on Integrated Plant Nutrition System and validated. We found that 0.38, 0.29, and 0.46 kg of N, P_2_O5, and K_2_O, respectively, were required for producing one quintal of beetroot tuber under the integrated approach. Readily customized of fertilizer nutrient doses was developed for varying soil test values and desired yield targets of beetroot, for both inorganic (NPK) alone and NPK + Farm Yard Manure (FYM). The model was validated in the same soil series with the achievement of 40 and 45 tonnes of beetroot ha^-1^ with 100.9% and 96.9% of yield achievement, respectively. The Soil analysis crop response - combined Plant Nutrition System model proved that beetroot yield can be increased by 34.74%, in relation to the generally recommended dose This inductive method could save 37, 26 and 34 kg of Nitrogen, Phosphorus and Potassium, respectively when Nitrogen, Phosphorus, Potassium fertilizers are combined with 12.5 t ha^-1^ FYM as per soil test and targeted yield of beetroot.

## 1 Introduction

Precision agriculture plays a vital role in fertilizer economy, plant yield productivity and soil health. Fertilizers are crucial for boosting agricultural production. Though fertilizer consumption over the years is increasing, achieving the fertilizer use efficiency at farm level is still in question due to variety of reasons. Continuous escalation of expense of fertilizers and limited quantity of organic manures left the farmers mainly rely on chemical fertilizers. The current blanket fertilizer recommendation in developing countries fails to ensure efficient and economical fertilizer use due to its oversight of fertility variations, leading to imbalanced utilization of fertilizer nutrients. Constant use of straight fertilizers resulted in deficiency of micronutrients in crops. The scientific community realized depending solely of chemical fertilizer or organic manure cannot sustain the crop productivity and soil health. Considering the fragmented small and marginal farms, soil test based fertilizer application found the most suited fertilizer recommendation model for country like India. Fertilizer recommendations based on soil test result in effective fertilizer use and sustain of soil fertility. Among the different techniques of fertilizer recommendation, the approach based on yield aimed stands out for its uniqueness, as it not only provides soil analysis based fertilizer doses but also predicts the achievable yield(production) level through appropriate crop management practices. The aimed yield methods also establishes a scientific foundation for equitable fertilization, ensuring equilibrium not only between nourishment from external input but also with those present in the soil.

The STCR method aids farmers in lowering fertilizer consumption and minimizing environmental contamination by supplying crops with precisely the nutrients they require. It also boosts crop productivity and profitability by optimizing nutrient application and preventing nutrient shortages or surpluses. One of the precision agriculture techniques that relies on soil fertility information combined with plant nutrient requirements to maximize nutrient management and crop yields is the Soil Test Crop Response approach. The utilization of this information concerning soil fertility and the plant requirements may help alleviate over-fertilization by minimizing the quantities needed and reduce environmental pollution. It can also enhance crop yields and profitability in the light of optimizing nutrient management and limiting the deficiencies or excesses of nutrient. Further, precision agriculture technologies like variable rate application can make nutrient management even more accurate and efficient.

*Beta vulgaris* L. (Beetroot) belong to Chenopodiaceae family, an crucial root vegetable which contain minerals like magnesium, manganese, sodium, potassium, iron, and copper. Beetroot has a various therapeutic characteristics that can help prevent heart disease and certain malignancies. Beetroot has many beneficial compounds, including glycine, betaine, saponins, betacyanin, carotenoids, folates, betanins, polyphenols, and flavonoids. Beetroot, a significant root vegetable crop cultivated in 2164 hectares, 36260 tonnes and 16.75 t ha^-1^ of productivity in India. The study area is Nilgiris, which covers 507 hectares and produces 11,915 tonnes, resulting in a productivity of 23.50 t h^-1^ (Kadam et al., 2018). Given that beetroot productivity is below the global average, it is imperative to boost productivity through improved technologies. A unique inductive cum targeted yield approach proposed by Ramamoorthy et al. (1967) that considers soil nutrient availability and economic yield target of crops with an inclusive fertilizer and manure combination suits the present situation for India. Santhi et al. (2011) developed the fertilizer prescription equation for beetroot grown in Alfisols distributed in plains of Tamil Nadu while there is no comprehensive fertilizer prescription equation for beetroot grown in Ulitsols (hilly soils) of Tamil Nadu. Hence, the present experiment was carried out in red non calcareous soils of Typic Haplohmult of Nilgiris (Ooty), Tamil Nadu (TN), India aimed to formulate a balanced fertilizer schedule to enhance both productivity and sustaining soil health with fertilizer economy in hill beetroot cultivation system.

## 2 Materials and methods

### 2.1 Details of the experimental field

**Figure 1:**
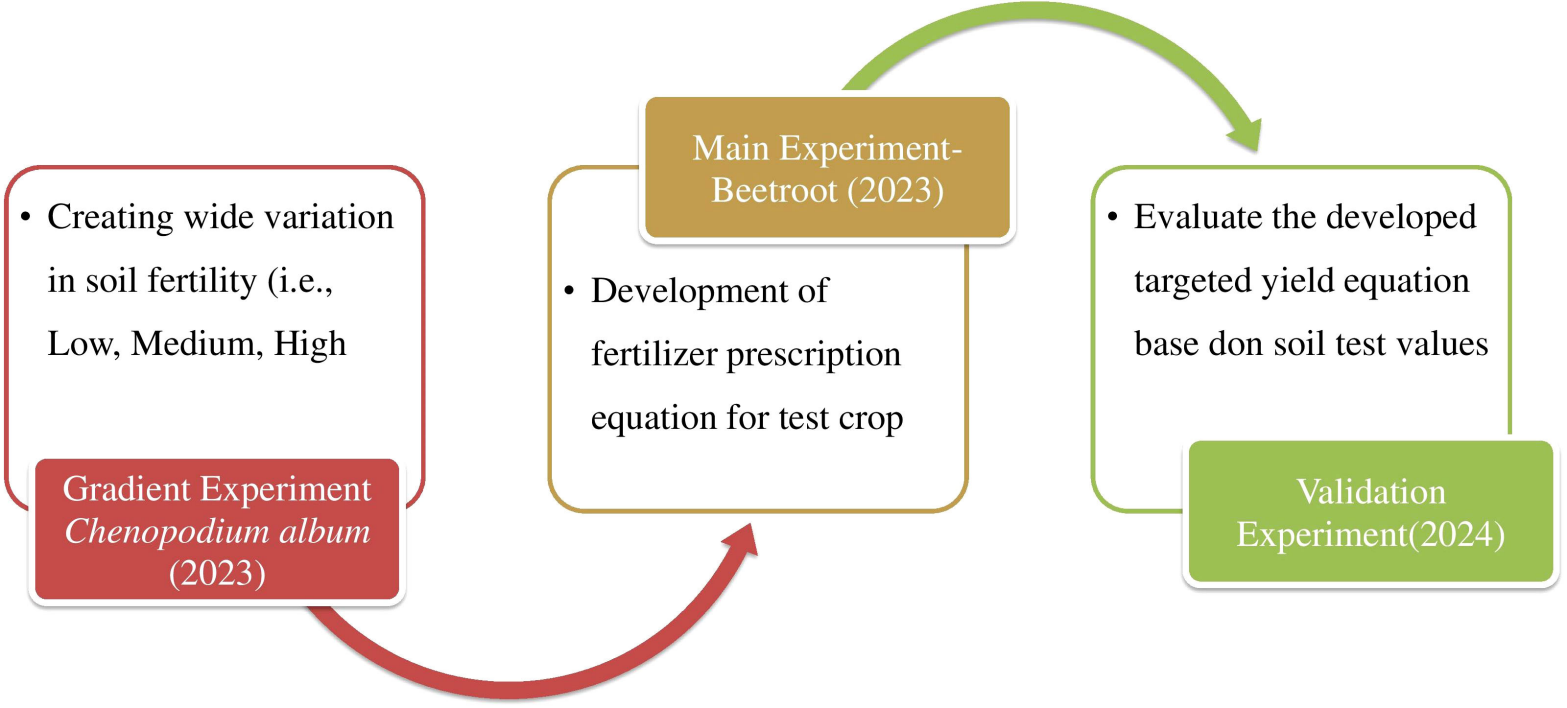
Graphical representation of experiment for development of fertilizer prescription equation.

Field trial were conducted in Typic Haplohmult soil series at Horticultural Research Station (HRS), a constituent research farm of Tamil Nadu Agricultural University (TNAU), in Nilgiris (11° 25’ N Latitude, 76° 43’ E Longitude), Tamil Nadu, India during 2023-2024. Surface (0-5.90 inch deep) soil sample was collected as per the standard procedure and analyzed for physical and chemical properties (Jackson, 1973). The fixing capacities of phosphorus were assessed using an equilibrium method with monocalcium phosphate, according to the procedure outlined by Waugh and Fitts (1966). Using an equilibrium technique using potassium chloride, the potassium fixation capacities were assessed in accordance with the protocol described by (Waugh and Fitts, 1966). The soil was red non calcareous, deep, well drained, clay loam in texture, acidic (pH 4.56), non-saline (EC 0.34 dSm^-1^) and medium in CEC (17 cmol (p+) kg^-1^). Initial soil fertility status indicated that the soil organic carbon, KMnO_4_-available nitrogen (N), Bray available phosphorus (P), NH_4_OAc- available potassium (K) were found to be 31.47 g kg^-1^, 450 kg ha^-1^, 185 kg ha^-1^, and 510 kg ha^-1^, respectively. The phosphorus and potassium fixing capacities of the initial soil were 250 and 100 kg ha^-1^, respectively. Available micronutrients (DTPA extractable- mg kg^-1^) viz., iron (42.14), Manganese (10.34), Zine(1.24) and Copper (1.82) were in sufficient ranges (**Figure 1**). **Figure 1** shows the overall protocol followed in the development STCR-IPNS model.

The field trial was carried out in three phase: Phase I: Fertility gradient field trial using a green, *Chenopodium album* from April to June 2023; Phase II: Test crop field trial with beetroot from September to December 2023; Phase III: Validation field trial with beetroot from January to April 2024 to confirm the accuracy of the developed targeted yield equation.

### 2.2 Fertility gradient field trial (phase I)

**Figure 2:**
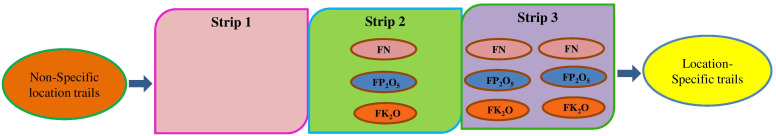
Illustration of the fertility gradient experiment.

First, a gradient experiment was conducted with a green, *Chenopodium album* var. Ooty 1. According to the inductive methodology proposed by Ramamoorthy et al. (1967), the fertility variation developed artificially. The experimental field trial was divided into three equal rectangular strips and applied with varying amounts of N, P_2_O_5_, and K_2_O fertilizers (N_0_P_0_K_0_; N_1_P_1_K_1_; N_2_P_2_K_2_). Strip I remained untreated nutrient (control), Strip II received the general recommendation of N recommended for *Chenopodium album*, while P_2_O_5_ and K_2_O were applied based on soil fixing capacities of 250 and 100 kg ha^-1^, respectively. Strip III received a double the dose of fertilizers compared to Strip II. Fertilizers were calculated, applied and ploughed which establishes a uniform mixing of fertilizers in each strip. After the ensuring mixing of fertilizers, *Chenopodium album* var. Ooty 1 was sown and irrigated. The intensive cultivation of *Chenopodium album* var. Ooty 1 led to alterations in the soil fertility. After 65 days of sowing, the crop was harvested in each fertility strip, and the yield of green biomass was recorded. Twenty four soil samples from each strip was collected and analyzed before and after the harvest of gradient crop for KMnO_4_ -nitrogen (N), Bray phosphorus (P) and ammonium acetate (NH_4_OAc) potassium (K). Based on the dry matter production, uptake of N, P and K, post harvest KMnO_4_-nitrogen (N), Bray phosphorus (P) and ammonium acetate (NH_4_OAc) potassium (K), the development of fertility gradient was ensured (**Figure 2**). **Figure 2** shows the layout of the gradient experiment and levels of nutrients added to each Strip.

### 2.3 Test crop field trail (phase II)

**Figure 3:**
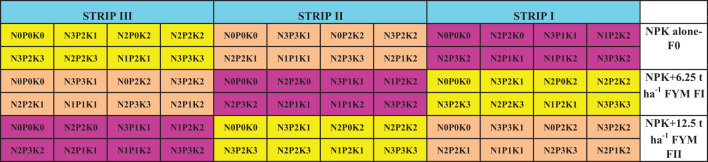
Layout plan of test crop experiment with Beetroot.

Second, a test crop experiment was planned with beet root (hybrid-Improved crystal). After the verification of gradient establishment, the principal experiment commenced with beetroot as the test crop. After the harvest of gradient crop, each strip was divided into 24 plots and soil samples from each plot was collected and analyzed for available potassium permanganate (KMnO_4_) nitrogen (N) (Subbiah and Asija, 1956), Bray phosphorus (P) (Bray and Kurtz, 1945), ammonium acetate (NH_4_OAc) potassium (K) (Stanford and English, 1949). The test crop experiment was laid out in a fractional factorial randomized block design comprising 24 treatments with four levels of N (0, 60, 120, and 180 kg ha^-1^), four levels of P_2_O_5_ (0, 80, 160, and 240 kg ha^-1^), four levels of K_2_O (0, 50, 100, and 150 kg ha^-1^) and three levels of FYM @ 0, 6.25, and 12.5 t ha^-1^. Within the field trial, each strip was subdivided into three sub-strips to apply three levels of FYM across the fertility gradient. The layout of the field trial is illustrated in **Figure 3** which shows the major nutrient combination for each treatment and superimposition of level of manure in each Strip main experiment. The test crop was cultivated using 24 treatment combinations by applying 21 selected treatment combinations, along with three control treatments as outlined in **Table 1**. Care was taken to super impose the IPNS treatments viz., NPK (inorganic) alone, NPK + FYM (integrated) at 6.25 t ha^-1^, and NPK + FYM (integrated) at 12.5 t ha^-1^) across the strips. Attention was ensured to randomize the 21 fertilizer treatments and three control treatments in such a way that all the 24 treatments present in all the three strips in either direction of the plots. All the nutrient management and plant protection packages were done as per the Crop Production Guide prescribed by Tamil Nadu Agricultural University and Government of Tamil Nadu. The fertilizer P_2_O_5_, K_2_O, and FYM were applied basally while fertilizer nitrogen (N) was applied during basal and 30 days after sowing. The crop was grown to maturity, fresh roots were harvested and the root yield was recorded. From each plot, plant and root samples were collected, processed, and analyzed for total N (Humphries, 1956), P, and K contents (Jackson, 1973), and the NPK uptake by beetroot was computed based on dry matter yield and NPK nutrient content.

**Table 1:** Treatment structure for test crop experiment (Beetroot).

S. No	Treatment combination			Levels of nutrients(kgha^-1^)		
	N	P	K	N	P_2_O_5_	K_2_O
1	0	0	0	0	0	0
2	0	0	0	0	0	0
3	0	0	0	0	0	0
4	0	2	2	0	160	100
5	1	1	1	60	80	50
6	1	2	1	60	160	50
7	1	1	2	60	80	100
8	1	2	2	60	160	100
9	2	1	1	120	80	50
10	2	0	2	120	0	100
11	2	1	2	120	80	100
12	2	2	2	120	160	100
13	2	2	1	120	160	50
14	2	2	0	120	160	0
15	2	2	3	120	160	150
16	2	3	2	120	240	100
17	2	3	3	120	240	150
18	3	1	1	180	80	50
19	3	2	1	180	160	50
20	3	2	2	180	160	100
21	3	3	1	180	240	50
22	3	3	2	180	240	100
23	3	2	3	180	160	150
24	3	3	3	180	240	150

The SPAD (Soil-Plant Analysis Development) meter value, which serves as an indirect measure of chlorophyll content in the leaf, was recorded at 30, 60, and 90 days after sowing (DAS) and utilized to calculate the chlorophyll concentration. This measurement acts as a diagnostic tool for assessing the nitrogen status of crops.

#### 2.3.1 Principal component analysis

Principal component analysis (PCA), as described by Wold et al. (1987), was employed to evaluate the potential impact of available soil nutrients, NPK fertilizers, and farmyard manures (FYM) on beetroot yield.

#### 2.3.2 Computation of basic parameters

Making use of the data on root yield, nutrient uptake, presowing soil available nutrients, and applied fertilizer doses, the basic parameters viz., nutrient requirement (NR) and contributions of nutrients from soil (C_s_) and contributions of nutrients from fertilizers (C_f_) were calculated as outlined by (Ramamoorthy et al., 1967; Velayutham et al., 1985; Krishna Murthy et al., 2023; Rao and Srivastava, 2000) and contributions of nutrients from Farm Yard Manure (C_fym_) were estimated as described by (Santhi et al., 1999). The nutrient requirement, soil, fertilizer, and farmyard manure efficiencies were derived as discussed by (MaruthiSankar, 1986). The basic parameters, NR expressed in kg per quintal while, C_s_, C_f_, and C_fym_, expressed as a percentage.


$$\text{F}=\frac{\text{NR}}{\text{Cf}}\text{Y}-\frac{\text{Cs}}{\text{Cf}}\text{S}-\frac{\text{CYFM}}{\text{Cf}}\text{FYM}$$

where F is fertilizer (kg ha^-1^), NR is nutrient requirement of N or P_2_O_5_ or K_2_O kg q^-1^ produce, Cs is percent contribution from soil, C_f_is percent contribution from fertilizer, C_FYM_ is percent contribution from FYM, S is soil-test value for available N, P, or K (kg ha^-1^), Y is yield target (q ha^-1^), and FYM is farmyard manure (t ha^-1^).

The relationship among soil test values, crop yield, and fertilizer dosage was determined using standard regression techniques, as outlined by (Draper and Smith, 1998). The current research investigates the fluctuation in beetroot yield resulting from the use of various fertilizer dosages, with other factors of soil and crop management practices are kept steady.

### 2.4 Validation experiment (phase III)

Third, a validation field trial was carried out in farmer’s land in same soil series, using beetroot (Hybrid: Improved Crystal) to verify the STCR-IPNS model. Based on the survey of the potential yield of the hybrid in Nilgiris, three beet root yield targets were taken (35.00 t ha^-1^, 40.00 t ha^-1^ and 45.00 t ha^-1^). This validation includes assessing the percentage achievement from the fixed target, beetroot yield, response ratio (RR), and economic comparisons with other fertilizer recommendation approaches such as the general fertilizer recommended dose, all within a RBD - randomized block design with three replication. Treatment consisted of T_1_-General fertilizer recommended dose (100% GFRD alone), T_2_-General fertilizer recommended dose (100% GFRD) + FYM @ 12.5 t ha^-1^, T_3_-STCR-Inorganic-TY_1_ 35.00 _t_ ha^-1^, T_4_-STCR-Inorganic-TY_2_ 40.00 _t_ ha^-1^, T_5_ - STCR-Inorganic-TY_3_ 45.00 _t_ ha^-1^, T_6_-STCR-Integrated-TY_1_ 35.00 _t_ ha^-1^, T_7_-STCR-Integrated-TY_2_ 40.00 _t_ ha^-1^ T_8_- STCR-Inorganic-TY_3_ 45.00 _t_ ha^-1^,T_9_- Farmer’s fertilizer Practice (FFP), T_10_-Absolute control (untreated nutrients). Composite soil samples were taken from each plot at a depth of 0-5.90inch before initiating the experiment, following the layout plan. Fertilizer application for STCR treatments (T_3_ to T_8_) was determined using STCR equations. The crop was cultivated according to standard crop production guide procedure, harvested at full maturity, and beetroot yields were computed based on the net plot area, expressed in tons per hectare (kg ha^-1^).

#### 2.4.1 Per cent achievement


$$\begin{array}{c} \text{Per cent achievement }\\ =\frac{\text{Yield obtained in the STCR treatment }\left({\text{kg or q ha}}^{–1}\right)}{\text{Yield targeted }\left({\text{kg or q ha}}^{–1}\right)}\times 100 \end{array}$$

#### 2.4.2 Response ratio


$$\begin{array}{c} \text{Response ratio}\\ =\frac{\text{Response }\left(\text{kg h}a^{–1}\right)}{{\text{Quantities of fertiliser N, P}}_{2}{\text{O}}_{5}{\text{and K}}_{2}\text{O applied }\left({\text{kg ha}}^{–1}\right)} \end{array}$$

## 3 Results

### 3.1 Fertility gradient field trial

A fertility gradient field trial was carried out to introduce variation in soil available NPK status across the trial field. **Table 2** illustrates the mean and range soil analysis values (STVs) for N, P_2_O_5_, and K_2_O, demonstrating the variation across fertility strips. Strip III exhibited the highest nutrient levels (467kg N, 213kg P_2_O_5_, and 620 kg K_2_O ha^-1^ respectively) due to the use of maximum fertilizers, compared to strip I (409 kg N, 178kg P_2_O_5_, and 509 kg K_2_O ha^-1^ respectively), and strip II (432 kg N, 194 kg phosphorus pentoxide (P_2_O_5_), and 550 kg potassium oxide (K_2_O) ha^-1^ respectively). Biomass yield of gradient crop (*Chenopodium album)* was also highest in strip III with 27.69 t ha^-1^, compared to strip II (24.00 t ha^-1^) and strip I (12.00 t ha^-1^).

**Table 2:** Descriptive statistics of Green biomass yield available soil nutrients (0-15 cm), after the soil fertility gradient experiment.

Strips		Soil available nutrients (kg ha^-1^)			Green Biomass yield (t ha^-1^)
		Nitrogen	Phosphorus	Potassium	
I	Range	395.00-424.00	170.40-184.94	487.62-532.46	11.82-12.36
	Mean ± SD	409 ± 11.10	178 ± 4.94	509 ± 4.60	12 ± 0.05
	(CV %)	2.70	2.77	2.56	1.33
	Median	412.50	178.63	509.99	11.99
II	Range	423.36-449.28	186.03-199.48	544.00-559.24	23.44-25.10
	Mean ± SD	432 ± 8.71	194 ± 4.29	550 ± 1.48	24 ± 0.17
	(CV %)	2.01	2.20	0.76	0.41
	Median	429.54	195.37	549.96	23.94
III	Range	445.00-482.00	209.00-224.00	610.08-639.84	27.35-29.28
	Mean ± SD	467 ± 11.10	213 ± 4.62	620 ± 3.18	28 ± 0.27
	(CV %)	2.37	2.16	1.45	0.64
	Median	469.50	213.00	619.00	27.69

SD, standard deviation; CV (%), coefficient of variation (%).

Strip I, Untreated nutrient (control); Strip II, General recommendation of N recommended for *Chenopodium album*, while P_2_O_5_ and K_2_O were applied based on soil fixing capacities of 250 and 100 kg ha^-1^, respectively; Strip III, Double the dose of fertilizers compared to Strip II.

### 3.2 Fertility strip variations influenced soil nutrient availability, beetroot yield, and nutrient uptake

The mean and range values of soil analysis results, nutrient uptake and beetroot yield for each strip is given in **Table 3**. The pre-sowing soil analysis values for beetroot indicated that the mean KMnO_4_- available nitrogen (N) was found to be 416, 435, and 475 kg ha^-1^ in strips I, II, and III, respectively. Similarly, the average pre-sowing soil analysis values for Bray-P were 184, 197, and 209 kg ha^-1^ in strips I, II, and III, respectively. Pre-sowing soil analysis values for NH_4_OAc-K were 515, 552, and 620 kg ha^-1^ in strips I, II, and III, respectively. The average values of KMnO_4_- available nitrogen (N), Bray-Phosphorus (P), and NH_4_OAc-available potassium (K) in inorganic treated plots were 442, 196, and 562 kg ha^-1^, respectively. In control plots, the average values of KMnO_4_- available nitrogen (N), Bray-Phosphorus (P), and NH_4_OAc-available potassium (K) were 438, 196, and 566 kg ha^-1^, respectively.

**Table 3:** Initial soil available NPK, as well as the yield and NPK uptake by beetroot, were recorded in various strips of the test crop experiment (kg ha^-1^).

Parameters (kg ha^-1^)	Strip I		Strip II		Strip III		Overall			
							NPK treated		Control (NPK)	
	Range	Mean	Range	Mean	Range	Mean	Range	Mean	Range	Mean
KMnO_4_-N	411-421	416	431-441	435	471-481	475	411-481	442.33	411-473	438.89
Bray-P	182.3-186.2	184.3	195.2-200.0	197.4	207.1-211.5	209.4	182.3-211.5	197.0	182.2-210.0	196.3
NH_4_OAc-K	510-519	515	547-557	552	615-625	620	510-625	562.00	517-625	566.00
Tuber Yield	10040-50835	33500	15545-58045	38950	18311-61800	44833	18481-61800	41951	10040-28072	19099
N uptake	64.5-191.0	135.7	89.3-205.1	145.9	93.3-210.1	164.5	83.15-210.19	156.08	64.5-112.6	97.4
P uptake	12.9-69.9	41.0	21.3-83.1	49.3	23.5-85.1	55.9	22.94-85.04	52.45	12.9-28.5	23.1
K uptake	99.5-161.0	135.5	123.5-175.2	149.4	125.4-220.2	168.1	108.03-220.22	155.43	99.5-138.4	120.54

Strip I: Untreated nutrient (control); Strip II: General recommendation of N recommended for *Chenopodium album*, while P_2_O_5_ and K_2_O were applied based on soil fixing capacities of 250 and 100 kg ha^-1^, respectively; Strip III: Double the dose of fertilizers compared to Strip II.

In strip I, the beetroot yield varied from 10,040 to 50,835 kg ha^-1^, with a mean (average) of 33,500 kg ha^-1^; in strip II, it ranged from 15,545 to 58,045 kg ha^-1^, with average of 38,950 kg ha^-1^; and in strip III, it ranged from 18,311 to 61,800 kg ha^-1^, with average of 44,833 kg ha^-1^. The average beetroot yield in inorganic treated plots and untreated nutrient plots were 41,951 kg ha^-1^ and 19,099 kg ha^-1^, respectively, representing a percentage increase of 119.65% over the control. The nutrient uptake data indicated variations in N uptake ranged from 64.5 to 210.1 kg ha^-1^, P uptake from 12.9 to 85.1 kg ha^-1^, and K uptake from 99.5 to 220.2 kg ha^-1^ across strips I, II, and III, respectively. The overall average values of nitrogen (N), phosphorus (P), and potassium (K) uptake in inorganic treated plots were 156.0, 52.4, and 155.4 kg ha^-1^, respectively. Within the control plots, the overall mean values for nitrogen (N), phosphorus (P), and potassium (K) uptake were 97.4, 23.1, and 120.5 kg ha^-1^, respectively.

### 3.3 Potential of available nutrients, Nitrogen, Phosphorus, Potassium fertilizers, FYM in beetroot yield production

**Figure 4:**
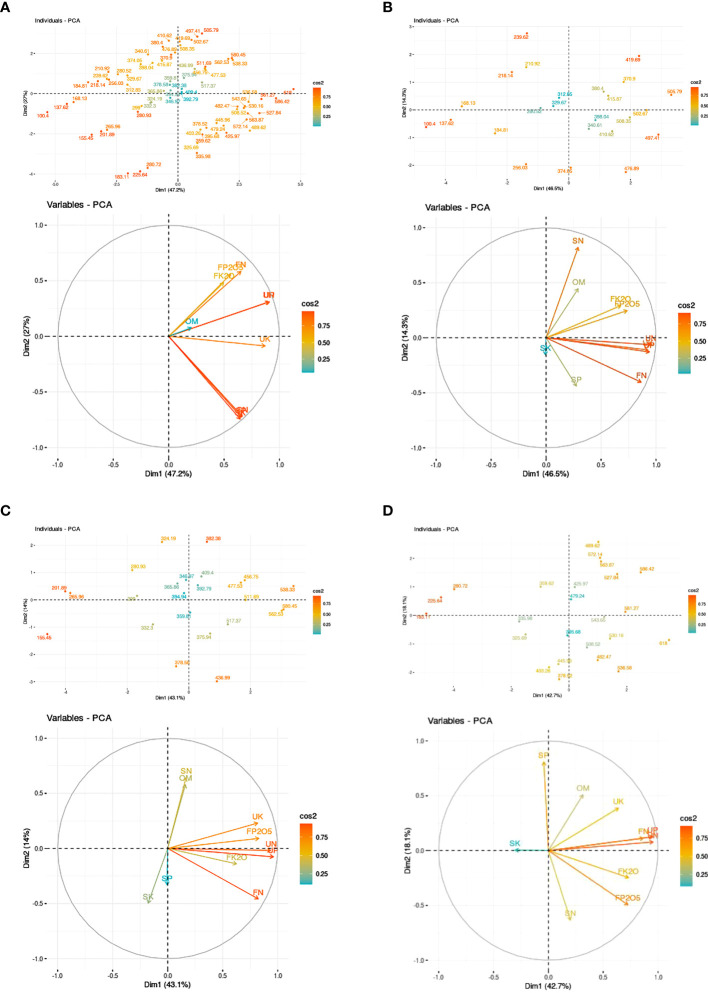
**(A)** Principle component analysis plots of overall relating the beetroot yield and the active variables. (i) Scoring plot showing the positions of each treatment. (ii) Loading plot showing the orthogonal positions of variable (Soil available nutrient (kg ha^-1^), NPK fertilizer (kg ha^-1^), FYM application (t ha^-1^), and NPK uptake by plant (kg ha^-1^). **(B)** Principle component analysis plots of strip 1 relating the beetroot yield and the active variables. (i) Scoring plot showing the positions of each treatment. (ii) Loading plot showing the orthogonal positions of variable [Soil available nutrient (kg ha^-1^), NPK fertilizer (kg ha^-1^), FYM application (t ha^-1^), and NPK uptake by plant (kg ha^-1^)]. **(C)** Principle component analysis plots of strip 2 relating the beetroot yield and the active variables. (i) Scoring plot showing the positions of each treatment. (ii) Loading plot showing the orthogonal positions of variable [Soil available nutrient (kg ha^-1^), NPK fertilizer (kg ha^-1^), FYM application (t ha^-1^), and NPK uptake by plant (kg ha^-1^)]. **(D)** Principle component analysis plots of strip 3 relating the beetroot yield and the active variables. (i) Scoring plot showing the positions of each treatment. (ii) Loading plot showing the orthogonal positions of variable [Soil available nutrient (kg ha^-1^), NPK fertilizer (kg ha^-1^), FYM application (t ha^-1^), and NPK uptake by plant (kg ha^-1^)].

Principal component analysis (PCA) was conducted for each strip to analyze the relationship between beetroot yield production and various factors, including available soil nutrients, N,P,K uptake, applied NPK chemical fertilizers, and FYM. **Figure 4A** presents the PCA plot illustrating the variables and observations for all strips. The PCA explained an average cumulative variability of 60.80% in beetroot production across all strips. This variability was attributed to 24 distinct combinations of NPK and FYM treatments under varying soil fertility variation. PCA analysis showed that among the variables assessed, application of NPK mineral fertilizer, NPK nutrient uptake, and organic manure (FYM) were consistently located orthogonally in the positive (+) quadrant (PC1 and PC2) across all strips. This positioning indicates that these variables made a significant positive (+) contribution to beetroot yield production.

PCA showed that PC1 of strip 1 comprised for 46.5% of the total cumulative variability. PCA analysis indicated that all evaluated variables were located orthogonally in the positive (+) quadrant (PC1 and PC2) (**Figure 4B**). Likewise, in strip 2 [available soil potassium (K)] and Strip 3 [available soil nitrogen (N) and potassium (K)], these variables were found in the negative (-) quadrant. This positioning may indicate lower nutrient contributions from the higher gradient soil towards beetroot yield.

In relation to those plots, the optimal sets of potential treatments were located in the “high” (+,+) quadrant and “moderate” (+,-) quadrants of the PCA plot (**Figures 4C, D**). Conversely, the treatment that exhibited lower potential in beetroot production revealed negative (-) correlation with the variables and was positioned in the “low” (-,-) and (-,+) quadrants.

Multiple regression equations were derived using variable as an independent variable (UN,UP,UK,SN,SP,SK,FN,FP_2_O_5_,FK_2_O,FYM) and the dependent variable yield was derived for each strip separately and as a whole. The relationship between yield and the variables was determined as follows:


(1) $$\begin{array}{c} Yield\;in\;stripI=-1264.03+(0.687\times UN)+(0.47\times UP)\\ +(1.046\times UK)-(1.084\times SN)+(3.135\\ \times SP)+(2.056\times SK)+(0.881\times FN)\\ +(0.169\times FP_{2}O_{5})+(0.133\times FK_{2}O)\\ +(4.825\times FYM) \end{array}$$

(2) $$\begin{array}{c} Yield\;in\;strip\;II\\ =-799.56+(0.794\times UN)+(0.968\times UP)+(0.642\times UK)\\ +(2.014\times SN)-(9.11\times SP)+(3.081\times SK)+(0.772\\ \times FN)+(0.060\times FP_{2}O_{5})+(0.398\times FK_{2}O)+(4.499\\ \times FYM) \end{array}$$

(3) $$\begin{array}{c} Yield\;in\;strip\;III\\ =622.634+(0.509\times UN)+(2.605\times UP)+(0.111\times UK)\\ -(0.336\times SN)+(1.603\times SP)(1.155\times SK)+(0.683\\ \times FN)+(0.141\times FP_{2}O_{5})+(0.176\times FK_{2}O)+(1.851\\ \times FYM) \end{array}$$

(4) $$\begin{array}{c} Yield\;in\;overall\;strips\\ =-211.45+(0.932\times UN)+(1.171\times UP)+(0.643\times UK)\\ -(0.892\times SN)+(0.582\times SP)+(0.816\times SK)+(0.679\\ \times FN)+(0.183\times FP_{2}O_{5})+(0.168\times FK_{2}O)+(3.026\\ \times FYM) \end{array}$$

The coefficient of determination (R²) values were ≥0.97 for each strip, as well as for the overall strip collectively. The results from multiple regression equations served as corroborating data for the PCA analysis.

### 3.4 Chlorophyll content

**Figure 5:**
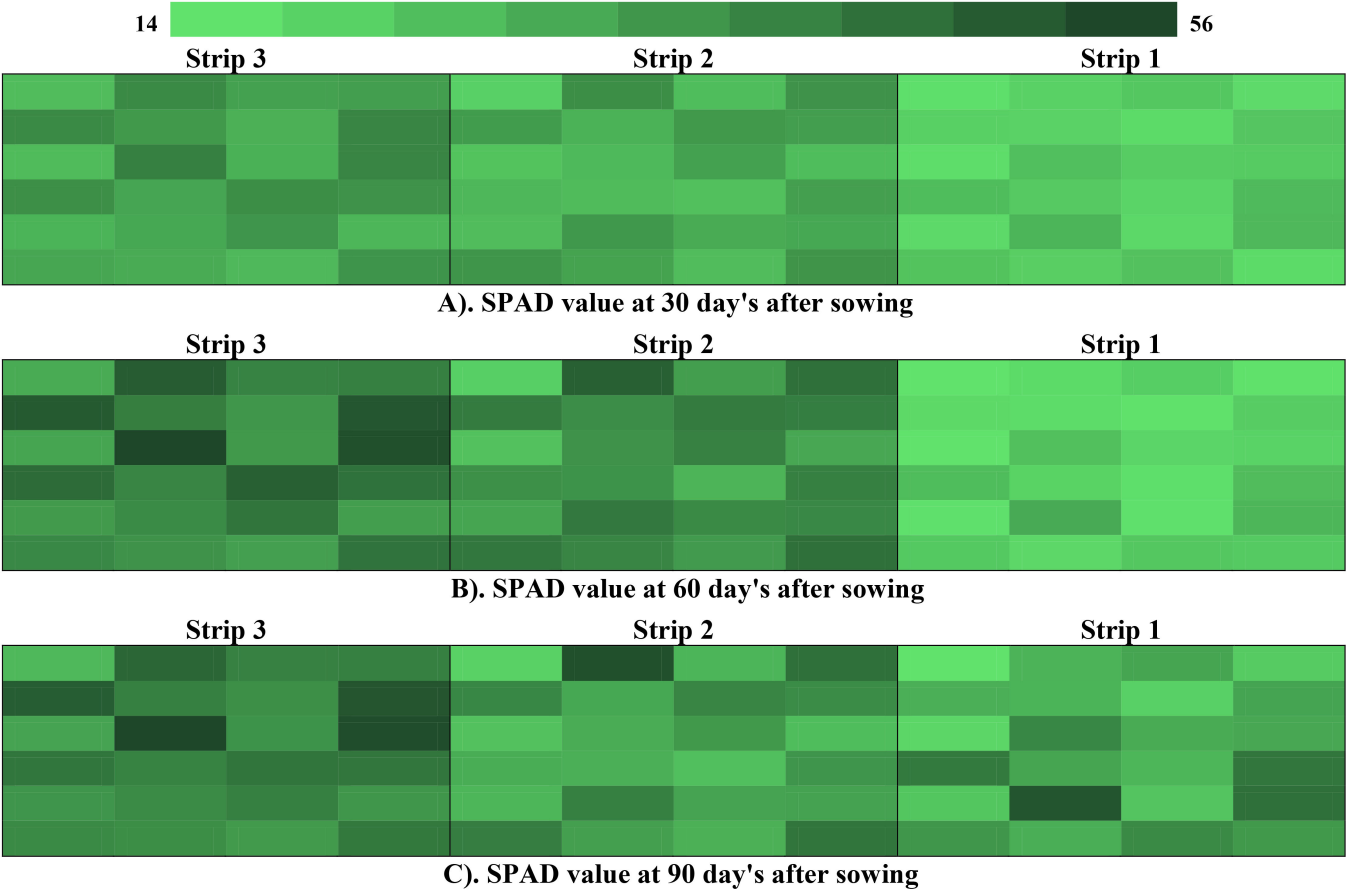
**(A)** SPAD value at 30 day’s after sowing. **(B)** SPAD value at 60 day’s after sowing. **(C)** SPAD value at 90 day’s after sowing.

The SPAD meter data ranged from 14.5 to 42.6 at 30 DAS, 24.0 to 55.9 at 60 DAS, and 29.6 to 61.9 at 90 DAS. The data indicated a gradual upward trend in chlorophyll content, with the mean values rising from 27.73 at 30 DAS to 45.51 at 90 DAS (**Figures 5A-C**).

### 3.5 Basic parameters

In the targeted yield model, the basic parameters were computed using data on beetroot yield, initial soil analysis values, NPK uptake and the amounts of applied N, P_2_O_5_, and K_2_O. The basic parameters for developing fertilizer prescription equations for beetroot are (i) nutrient requirement in kg per quintal of beetroot (NR) and percentage contributions from soil-available nutrients (C_s_), fertilizer nutrients (C_f_), and Farm yard manure (C_fym_) are given in **Table 4**.

**Table 4:** Nutrient requirement and contributions of nutrients from soil, fertilizer, and FYM for beetroot.

Parameters	Nutrients		
	N	P_2_O_5_	K_2_O
Nutrient requirement (kg q^-1^)	0.38	0.29	0.46
Per cent contribution from soil (C_s_) (%)	20.25	11.02	19.67
Per cent contribution from fertilizers (C_f_) (%)	55.16	46.62	56.62
Per cent contribution from FYM (C_fym_) (%)	34.40	17.46	29.65

### 3.6 Nutrient requirement

**Figure 6:**
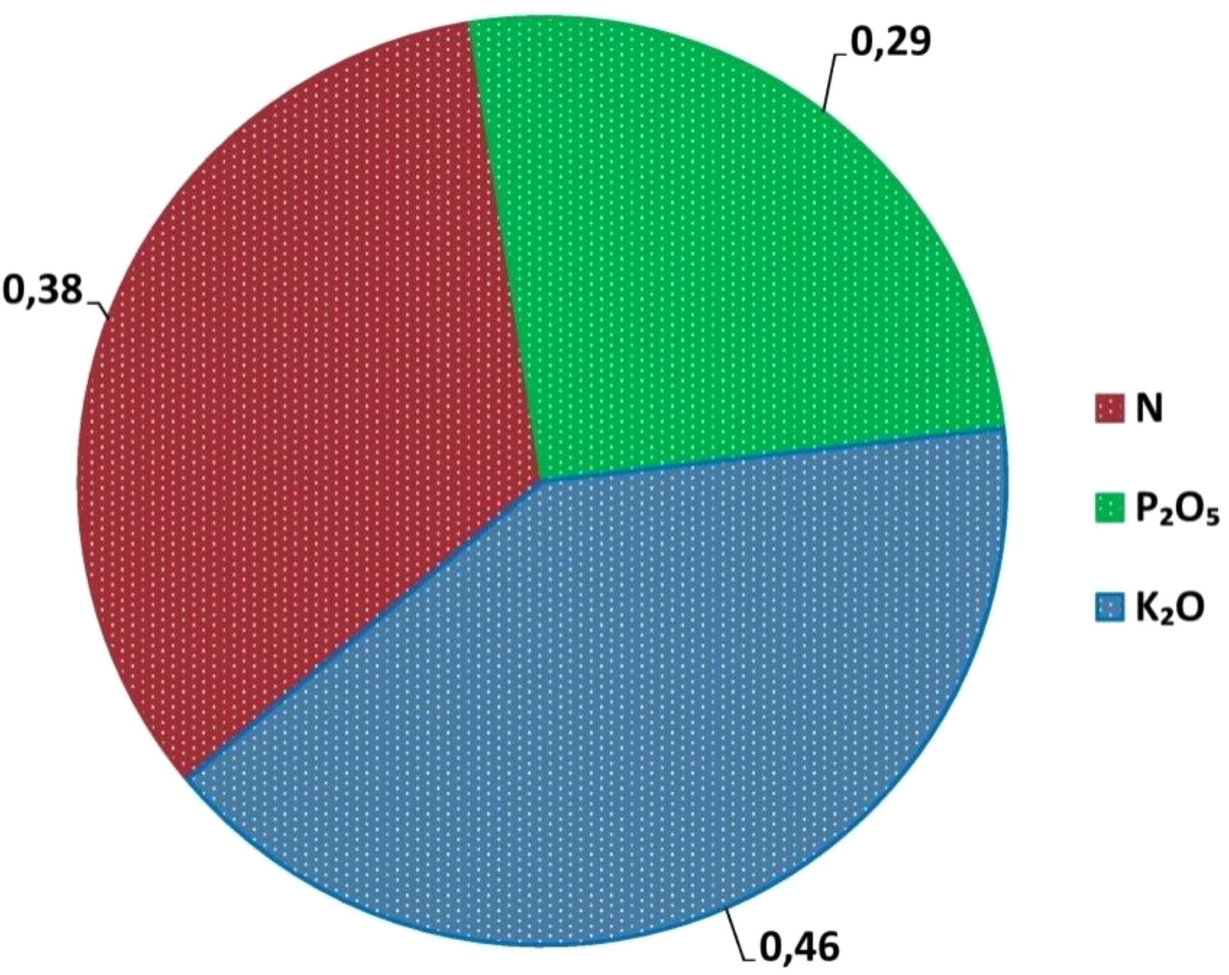
Nutrient requirement (kg q^-1^) of N, P_2_O_5_ and K_2_O for Beetroot.

The nutrient requirement (NR) is defined as the amount of nutrient needed to produce a single unit of economic yield. To produce 100 kg of beetroot yield, the required amounts of nutrients were found to be 0.38 kg of N, 0.29 kg of P_2_O_5_, and 0.46 kg of K_2_O (**Figure 6**).

### 3.7 Percentage contributions of nutrients from soil, fertilizers, and farm yard manure to total uptake

The soil’s available nutrient contribution (Cs) was reported as 20.25% for N, 11.02% for P_2_O_5_, and 19.67% for K_2_O. The percentage contribution of N, P_2_O_5_, and K_2_O from fertilizers (C_f_) was 55.16, 46.62, and 56.62, respectively, following the order of K_2_O > N > P_2_O_5_. The organic manure (FYM) contribution (C_fym_) was recorded as 34.40% for N, 17.46% for P_2_O_5_ and 29.65% for K_2_O, respectively (**Table 4**).

### 3.8 Fertilizer prescription equations

The beetroot yield target model was developed based on basic parameters.

**STCR-Inorganic equation**


(5) $$\;\text{FN }=0.69\text{ T}-0.37\text{ SN }\left({\text{KMnO}}_{4}-\text{N}\right)$$

(6) $${\text{FP}}_{2}{\text{O}}_{5}=0.61\text{ T}-0.54\text{ SP }\left(\text{Bray}-{\text{P}}_{2}{\text{O}}_{5}\right)$$

(7) $${\text{FK}}_{2}\text{O }=0.82\text{ T}-0.42\text{ SK }\left(\text{Am. Ace.}-{\text{K}}_{2}\text{O}\right)$$

**STCR-IPNS equation**


(8) $$\text{FN }=0.69\text{ T}-0.37\text{ SN}\left({\text{KMnO}}_{4}-\text{N}\right)-0.62\text{ ON}$$

(9) $${\text{FP}}_{2}{\text{O}}_{5}=0.61\text{ T}-0.54\text{ SP}\left(\text{Bray}-{\text{P}}_{2}{\text{O}}_{5}\right)-0.86\text{ OP}$$

(10) $${\text{FK}}_{2}\text{O }=0.82\text{ T}-0.42\text{ SK}\left(\text{Am. Ace}.-{\text{K}}_{2}\text{O}\right)-0.63\text{ OK}$$

where FN, FP_2_O_5_, and FK_2_O are fertilizer N, P_2_O_5_, and K_2_O in kg ha^-1^, respectively; T is the yield target in q ha^-1^; SN, SP, and SK are available soil nutrients as KMnO_4_-N, Bray’s-P_2_O_5_, and NH_4_OAc-K_2_O in kg ha^-1^, respectively, and ON, OP, and OK are the quantities of Nitrogen, Phosphorus, and Potassium supplied through farmyard manure in kg ha^-1^

### 3.9 Fertilizer prescription under integrated for desired yield target of beetroot

A readily available table was developed according to these equations for diverse soil test values and a yield target of 400 q ha^-1^ (**Table 5**). The findings clearly revealed that the fertilizer requirements for N, P_2_O_5_, and K_2_O decreased as soil analysis values increased.To attain a yield goal of 400 q ha^-1^ of beetroot with soil analysis values of 400 kg ha^-1^ for KMnO_4_-N, 180 kg ha^-1^ for Bray P, and 520 kg ha^-1^for NH_4_OAc-K, the required fertilizer doses were 128 kg ha^-1^of N, 147 kg ha^-1^of P_2_O_5_, and 110 kg ha^-1^of K_2_O, respectively. When farmyard manure (containing 26% moisture and 0.56%, 0.23%, and 0.47% of N, P, and K, respectively) was applied at a rate of 12.5 t ha^-1^, along with NPK, the required fertilizer doses were 91 kg ha^-1^ of N, 121 kg ha^-1^of P_2_O_5_, and 76 kg ha^-1^of K_2_O, respectively. Under IPNS, the savings in fertilizer were 18 kg ha^-1^of N, 13 kg ha^-1^of P_2_O_5_, and 17 kg ha^-1^of K_2_O when using NPK plus FYM at 6.25 t ha^-1^, and 37 kg ha^-1^of N, 26 kg ha^-1^of P_2_O_5_, and 34 kg ha^-1^of K_2_O when using NPK plus farmyard manure at 12 t ha^-1^, respectively.

**Table 5:** Reduction of inorganic fertilizers when soil-test-based fertilizer prescription under integrated (IPNS) for 400 q ha^-1^ target yield of beetroot (kg ha^-1^).

Parameter	NPK alone (kg ha^-1^)	NPK+FYM @6.25 t ha^-1^	Reduction over NPK alone (%)	NPK+FYM @12.5 t ha^-1^	Reduction over NPK alone (%)
KMnO_4_-N (kg ha^-1^)					
400	128	110	14.1	91	28.9
420	121	102	15.7	84	30.6
440	113	95	15.9	77	31.9
460	106	88	17.0	69	34.9
480	98	80	18.4	62	36.7
500	91	73	19.8	60*	34.1
Bray-P (kg ha^-1^)					
180	147	134	8.8	121	17.7
190	141	128	9.2	115	18.4
200	136	123	9.6	110	19.1
210	131	118	9.9	104	20.6
220	125	112	10.4	99	20.8
230	120	107	18.8	94	21.7
NH_4_OAc-K (kg ha^-1^)					
520	110	93	15.5	76	30.9
540	101	84	16.8	68	32.7
560	93	76	18.3	59	36.6
580	84	68	19.0	51	39.3
600	76	59	22.4	50*	34.2
620	68	51	25.0	50*	26.5

### 3.10 Validation experiment

#### 3.10.1 Beetroot yield

In our research, notable variation in beetroot yield was recorded across various treatments, lowest yield being 24.96 t ha^-1^ in the absolute control (untreated nutrient) to 43.60t ha^-1^, the highest yield, achieved by the STCR- integrated (IPNS)-45.00 t ha^-1^ treatment, as outlined in **Table 6**, exceeding all other treatments.

**Table 6:** Influence of different approaches of nutrient recommendations on yield, per cent achievement, Response Ratio (RR) and Benefit Cost ratio of beetroot crop.

S.No	Treatments	FYM (t ha^-1^)	Fertilizer doses (kg ha^-1^)			Beetroot Yield (t ha^-1^)	Per cent achievement	RR (kg kg^-1^)	BCR
			FN	FP_2_O_5_	FK_2_O				
1	T_1_	–	120	160	100	28.45	–	9.19	2.35
2	T_2_	12.5	120	160	100	29.46	–	11.85	2.41
3	T_3_	–	123	141	89	35.45	101.3	29.72	2.42
4	T_4_	–	172	201	128	39.40	98.5	28.81	2.53
5	T_5_	–	180**	240**	150**	43.42	96.5	32.39	2.63
6	T_6_	12.5	91	114	50*	36.22	103.5	44.16	2.44
7	T_7_	12.5	140	174	77	40.36	100.9	39.37	2.55
8	T_8_	12.5	180**	233	116	43.60	96.9	35.24	2.66
9	T_9_	–	100	120	80	26.42	–	4.86	1.74
10	T_10_	–	0	0	0	24.96	–	–	1.38

*Maintenance dose **maximum dose.

T_1_-General fertilizer recommended dose (100% GFRD alone), T_2_-General fertilizer recommended dose (100% GFRD) + FYM @ 12.5 t ha^-1^, T_3_-STCR-Inorganic-TY_1_ 35.00 _t_ ha^-1^, T_4_-STCR-Inorganic-TY_2_ 40.00 _t_ ha^-1^, T_5_ - STCR-Inorganic-TY_3_ 45.00 _t_ ha^-1^, T_6_-STCR-Integrated-TY_1_ 35.00 _t_ ha^-1^, T_7_-STCR-Integrated-TY_2_ 40.00 _t_ ha^-1^ T_8_- STCR-Inorganic-TY_3_ 45.00 _t_ ha^-1^,T_9_- Farmer’s fertilizer Practice (FFP), T_10_-Absolute control (untreated nutrients).

#### 3.10.2 Per cent achievement

The effectiveness of fertilizer recommendation calculations relies on the percentage attainment falling within the ±10% range scope of the yield target. In this context, the percentage achievement varied from 96.5% in STCR-inorganiconly at 45 t ha^-1^ to 103.5% in STCR-integrated at 35 t ha^-1^, indicating the applicability of the inductive model for beetroot across all three yield target tiers within both the STCR-inorganic only and integrated categories. In the STCR- integrated category, the higher yield target attainment was recorded in STCR-integrated-35 t ha^-1^ (103.5%), followed by STCR-inorganic alone 35 t ha^-1^ (101.3%) and STCR- integrated 40 t ha^-1^(100.9%). Conversely, in the case of STCR- inorganiconly, the percentage achievement for yield targets of 35, 40, and 45 t ha^-1^ was 103.5%, 100.9%, and 96.9%, respectively (**Table 6**).

#### 3.10.3 Response ratio and B:C ratio

The response ratio observed for different treatments varied from 4.86 kg kg^-1^ in farmer’s fertilizer practice to 44.16 kg kg^-1^ in STCR-integrated-35 t ha^-1^, followed by STCR-integrated-40 t ha^-1^ (39.37), STCR-integrated-45 t ha^-1^ (35.24 kg kg^-1^), and STCR-inorganiconly 45 t ha^-1^ (32.39 kg kg^-1^) (**Table 6**). Among the STCR treatments, STCR- integrated consistently showed highest response ratios compared to their respective STCR-inorganic only treatments. The general fertilizer recommended dose (100% GFRD alone), and the general fertilizer recommended dose (100% GFRD) + FYM @ 12.5 t ha^-1^ recorded response ratios of 9.19 and 11.85 kg kg^-1^, respectively, which were comparatively lower than all STCR treatments. According to the BCR data, STCR- integrated-45 t ha^-1^ (02.66) exhibited the highest value, followed by STCR-inorganic alone - 45 t ha^-1^ (2.63).

## 4 Discussion

The statistical evaluation revealed that every strip differs significantly from the others, and the use of varying levels of NPK fertilizers led to a notable rise in the soil’s available N, P, and K content, demonstrating the establishment of soil fertility variations in the field trial. Therefore, the establishment of soil fertility variation was verified by the soil analytical data for all three essential nutrients. The statistical analysis of post-harvest soil test data highlighted that significant difference in soil fertility status were present among the three strips (Udayakumar and Santhi, 2017; Singh et al., 2020; Vamshi et al., 2023). Using a graded level of fertilizer on gradient crops of rice, researchers found that grain and straw yields were higher. This could be because of the increased availability of nutrients in the soil with the increased levels of N, P, and K fertilizers, as well as the positive effects of these nutrients on fodder crops (Verma et al., 2014).

The experimental data clearly indicated significant variations in soil analysis value before sowing, nutrient uptake, and beetroot yield among the fertility strips and between NPK treated and control plots (untreated nutrient). These variations are crucial for establishing soil fertility gradients and are necessary for computing basic parameters and formulating fertilizer recommendation equations. These results align with the previous findings of (Umadevi, 2005; Singh, 2021) for carrot and Smitha John, 2004) for cabbage. The PCA results indicated that all variables were located in the positive quadrant, indicating their high importance for beetroot yield production. This finding aligns with the conclusions of a study by (Abishek et al., 2023).

**Figure 7:**
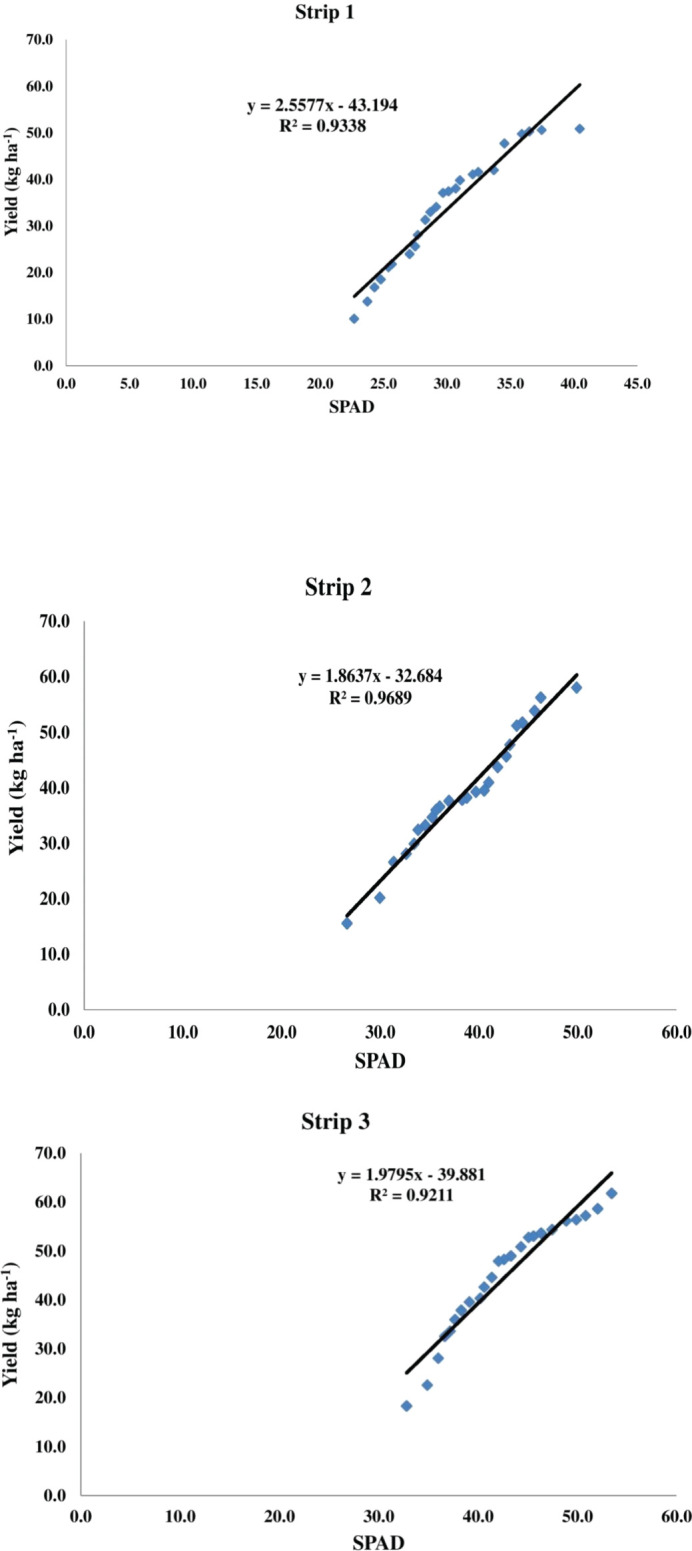
Linear regression of yield and chlorophyll content.

SPAD value(chlorophyll content) increase may be due to the readily available micro and macronutrients, especially nitrogen, provided by farm yard manure (FYM), which is a crucial component of chlorophyll. These results are consistent with the findings of (Dhakal et al., 2009) in spinach beet. The application of farm yard manure (FYM) may have enhanced microbial activity in the root zone of the beetroot plant, facilitating nutrient transformation. These findings are consistent with the experiments conducted by (Tovihoudji et al., 2015: Singh et al., 2017; Dhakal et al., 2016; Nagar et al., 2016). Chlorophyll exhibited a positive (+) correlation with beetroot yield **Figure 7**, with r values of strip 1 (0.93), strip 2 (0.96), and strip 3 (0.92). Similar results were reported in beetroot (Mounika et al., 2022; Kumar et al., 2023).

The nutrient requirements data indicated that the hierarchy of nutrient requirements was K_2_O >N > P_2_O_5_(descending order). Similar findings were observed by (Santhi et al., 2011 in beetroot; Kaushik et al., 2015 in radish; Dibyendu et al., 2010 in potato; KasthuriThilagam and Natesan, 2009 in cauliflower). Among the three essential nutrients, the soil’s contribution is highest for N, followed by K_2_O, and then P_2_O_5_. These findings closely align with those reported by (Gayathri et al., 2009) for Potato on Ultisols.

**Figure 8:**
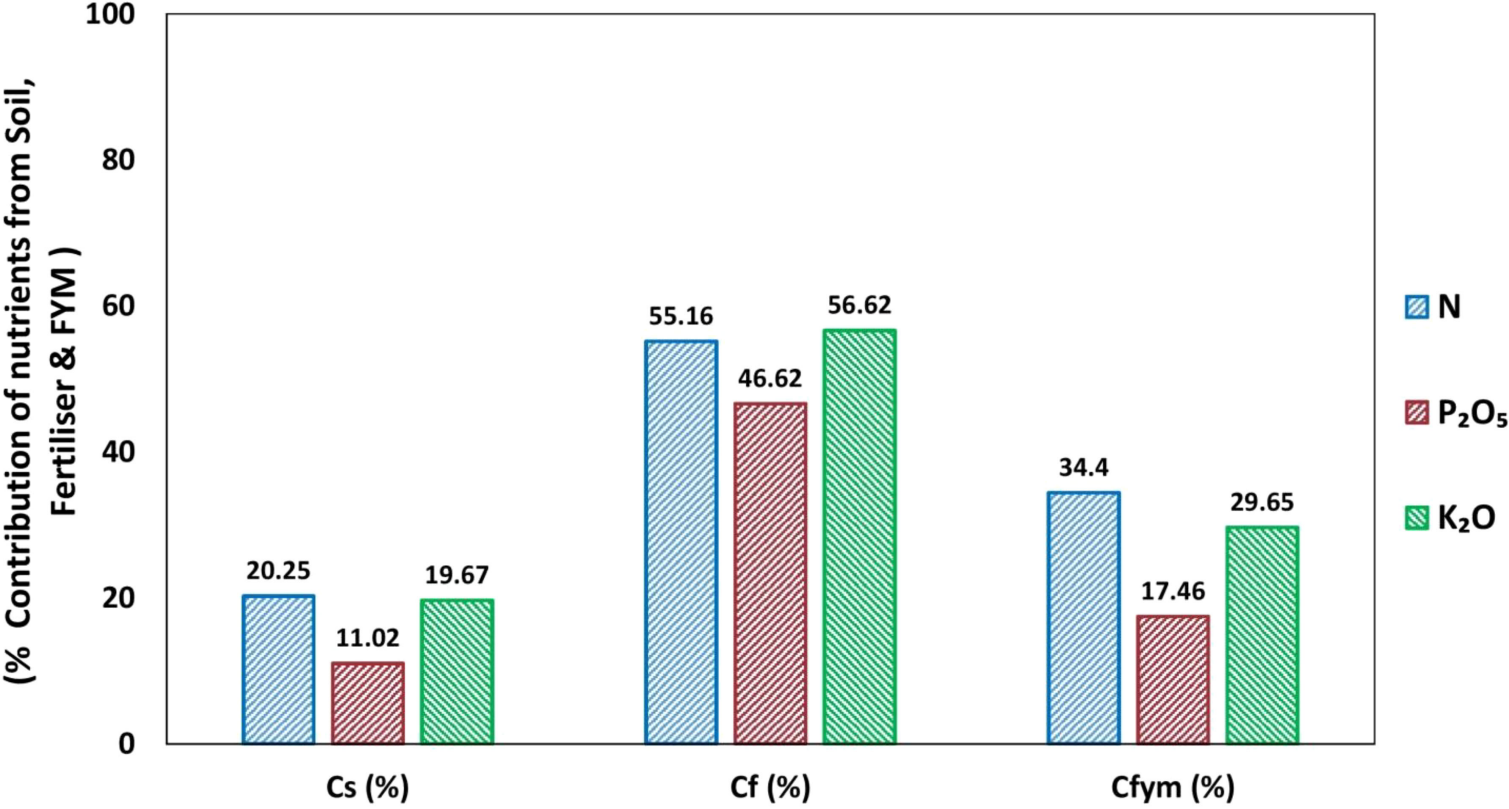
Contribution of Nutrient from Soil, Fertilizer and FYM(%).

The data indicated that the ranking of nutrient requirements was K_2_O > N > P_2_O_5_. The results are also consistent with the findings of (Santhi et al., 2011; Basavaraja et al., 2011; Polara et al., 2012; Sellamuthu et al., 2015; ParvathiSugumari et al., 2021; Bhavya et al., 2023) who observed a similar trend of relatively higher nutrient contribution of K_2_O compared to N and P_2_O_5_ from fertilizer. The organic manure (FYM) contributes more towards N (**Figure 8**). These results are consistent with the findings of (Gayathri et al., 2009; Anil et al., 2008; Singh et al., 2019). The basic parameters were computed based on the pre sowing soil test values, nutrient uptake and crop yield by beetroot. Using these basic parameters, fertilizer prescription equations were developed for beetroot on Ultisol to achieve precise beetroot yield targets.

Santhi and her colleague formulated fertilizer prescription equations and recorded them for various crops such as rice (Santhi et al., 1999), beetroot (Santhi et al., 2011), aggregatum onion (Santhi et al., 2005), and sunflower across different regions of Tamil Nadu.

The nutrient amounts can be subtracted from the recommended fertilizer quantity based on specific soil analysis values and yield targets. Kaushik et al. (2015) also reported that applying 12.5 t ha^-1^ of farmyard manure along with chemical fertilizer resulted in savings of 15 kg ha^-1^of N, 1.8 kg ha^-1^of P_2_O_5_, and 5 kg ha^-1^of K_2_O in radish. Comparatively, when inorganic fertilizer was applied alongside FYM, there was a significant reduction in fertilizer amounts compared to applying inorganic fertilizer alone, owing to the nutrient supplementation provided by FYM. There was an increasing percentage reduction in fertilizers when applied alongside FYM, with a corresponding increase in soil test values. Similar findings were reported by (Singh et al., 2018). Sellamuthu et al. (2019) also found that using 12.5 t ha^-1^ of farmyard manure in combination with chemical fertilizers saved 40 kg ha^-1^of N, 20 kg ha^-1^of P_2_O_5_, and 33 kg ha^-1^of K_2_O in big onion.

In contrast to STCR-NPK alone treatments with corresponding yield targets, STCR-IPNS treatments consistently exhibited higher yields, highlighting the beneficial synergy of combining inorganic fertilizers with organic manures. This combination demonstrated its superiority in enhancing crop productivity. The slow release of nutrient from FYM hindered its ability to sufficiently meet the essential nutrient needs during the crucial growth stages of the crop. The utilization of farmyard manure (FYM) in this context probably strengthened the nitrogen provision, thereby boosting beetroot production. Similar findings were reported by (Laharia et al., 2020; Zannat et al., 2020; Mohamed et al., 2023 and Anasuyamma et al., 2022).

Per cent achievement results suggest that utilizing IPNS for yield targeting consistently attained a greater percent of the desired target compared to employing inorganic alone treatments. This observation resonates with findings from a study conducted by (Dey and Bhogal, 2016; Santhi et al., 2017; Udayakumar and Santhi, 2016 for pearl millet; Abishek et al., 2022 on castor; Mohamed, 2023 for finger millet).

Benefit-Cost Ratio (BCR) of STCR- integrated (IPNS)was significantly greater than that of STCR-inorganic alone. The BCRs for the general fertilizer recommended dose (100% GFRD alone) and farmer’s fertilizer practice were 2.35 and 1.74, respectively, which were lower than all soil analysis crop response treatments. The fluctuations in benefit-cost ratios (BCRs) were mainly attributed to differences in crop yields and varying expenses associated with the use of farm farmyard manure (FYM). It is evident that the prudent utilization of organic inputs, like on FYM, alongside synthetic fertilizers, results in a more profitable outcome. Similar results were reported by (Lakum et al., 2011; Choudhary et al., 2014; Sipai et al., 2014; Singh and Chauhan, 2016; Meena et al., 2017; Raghav et al., 2019).

## 5 Conclusions

Blanket recommendation of fertilizers to crops leads to either overuse or under use of fertilizers. Soil test and yield targeting based STCR-IPNS approach by inductive methodology demonstrated that the fertilizer prescription to beetroot, enhances the beetroot yield. It has been clarified that STCR- Integrated (IPNS)provides a well-proportioned supply, accounting contribution from farmyard manure, soil and fertilizer, to achieve desired yield aimed of beetroot. When a farmer applying 12.5 t ha^-1^ of FYM, they can reduce 37, 26 and 34 kg of Nitrogen (N), Phosphorus (P) and Potassium (K), respectively from the prescribed dose of inorganic fertilizers. Under conventional recommendation, along with FYM, mineral fertilizers are applied without considering other sources. Findings from these experiments revealed that there is a significant response by beetroot to N, P and K fertilizers. Nutrient prescription using STCR-IPNS approach is able to achieve 100.9% and 96.9% yield targets of 40 t ha^-1^ (40.36 t ha^-1^) and 45 t ha^-1^ (43.60 t ha^-1^) respectively. The percentage attainment of the desired yield was within a ±10% deviation at yield target, confirming the accuracy of the fertilizer prescription model for recommending combined fertilizer (inorganic, organic) doses for beetroot. Though availability of the FYM is reducing day by day, explicit adoption of STCR-IPNS model will encourage the farmers to produce FYM at farm level and use it for sustaining soil health with higher economic returns.

## Data Availability

The raw data supporting the conclusions of this article will be made available by the authors, without undue reservation.
